# OCT Evaluation of Marginal and Internal Interface Integrity of Class V Composite Restorations after 36 to 48 Months

**DOI:** 10.3290/j.jad.b2916433

**Published:** 2022-04-13

**Authors:** Rainer Haak, Philip Schäfer, Bettina Hanßen, Dirk Ziebolz, Kyung Jin Park, Matthias Häfer, Gerhard Schmalz, Hartmut Schneider

**Affiliations:** a Professor and Chair, Department of Cariology, Endodontology and Periodontology, University of Leipzig, Germany. Idea, design, supervision of the study, discussed results, proofread the manuscript.; b Dentist in Private Practice, Berlin, Germany. Performed OCT analysis, contributed to statistical evaluation in partial fulfillment of requirements for a doctoral degree, wrote the manuscript.; c Dentist in Private Dental Practice, Hamburg, Germany. Contributed to statistical evaluation and discussion, proofread the manuscript.; d Associate Professor, Department of Cariology, Endodontology and Periodontology, University of Leipzig, Germany. Contributed to the discussion.; e Dentist, Department of Cariology, Endodontology and Periodontology, University of Leipzig, Germany. Performed OCT imaging, contributed to the discussion.; f Assistant Professor, Department of Cariology, Endodontology and Periodontology, University of Leipzig, Germany. Project coordinator, performed statistical evaluation, wrote the manuscript.; g Assistant Professor, Physicist, Department of Cariology, Endodontology and Periodontology, University of Leipzig, Germany. Design, project coordinator, performed statistical evaluation, co-wrote the manuscript.; * These authors contributed equally to this work.

**Keywords:** class V composite restoration, self-etching adhesive, etch-and-rinse adhesive, OCT, internal interfacial gap formation, quantitative margin analysis

## Abstract

**Purpose:**

To compare a self-etch and a two-step etch-and-rinse adhesive in terms of internal and marginal composite-tooth bond failure separately on enamel and dentin/cement at 36–48 months after restoration placement using optical coherence tomography (OCT).

**Materials and Methods:**

Twenty-seven patients with two or three class V composite restorations of noncarious cervical lesions 36–48 months after placement were included. The one-step self-etch adhesive Futurabond M ([Voco] group SE, n = 25) and the two-step etch-and-rinse adhesive Solobond M ([Voco] group ER, n = 20) combined with the nanohybrid composite Amaris (Voco) were evaluated. The four-step etch-and-rinse adhesive Syntac classic combined with Tetric EvoCeram (Ivoclar Vivadent) served as the control (n = 18). Spectral-domain OCT (SD-OCT, 1310-nm center wavelength) was applied. Marginal gaps and internal interfacial adhesive defects were quantified in cross-sectional OCT images. Groups were statistically compared using the Friedman/Wilcoxon test (α = 0.05).

**Results:**

In enamel, nonsignificantly different percentages of marginal gap formation and internal interfacial adhesive defects were found between the groups (p_i_ ≥ 0.258). In dentin/cement, SE showed significantly less marginal gap formation compared to ER (p < 0.001) and control (p = 0.001), and at the internal dentin-composite interface less adhesive defects were found compared to ER (p < 0.001) and control (p = 0.003).

**Conclusion:**

The self-etch adhesive used in the current study appears recommendable for restoration of noncarious cervical lesions with composite.

Adhesively luted restorations of noncarious class V cavities are a fundamental challenge in restorative dentistry. Due to the lack of macromechanical retention, a strong composite-tooth bond is demanded of the restorative systems for long-lasting clinical success. Moreover, the skills of the treating dentist and technical challenges, eg, moisture control or the limitation of the lesion to dentin, play a relevant role.^11,27^ Additionally, the type and brand of adhesive are important. Different adhesives are available, with a general distinction being made between etch-and-rinse and self-etch adhesives.^31^ Etch-and-rinse adhesives, which always include a separate step of etching/conditioning of the tooth surface, have been seen as the gold standard for many years.^14^ While a separate etching step is eminent for enamel, on dentin, self-etching without separate conditioning (phosphoric acid) might be superior and provide more predictable results in lesions exclusively in dentin.^17,21^ Moreover, self-etching adhesives might be easier to handle, be less technically sensitive during application, and offer advantages in dentin conditioning, such as avoiding over-etching with increased risk of collapse of collagen in dentin.^17^ Different clinical studies have been conducted which compared representatives of both adhesive classes regarding their clinical performance in noncarious class V restorations, primarily focusing on FDI criteria^12^ as clinical outcome parameters. A current meta-analysis concluded comparable clinical outcomes of both adhesive classes, whereby etch-and-rinse adhesives were slightly superior, with fewer discolorations of the restoration margin.^26^ Although clinical parameters, such as survival, clinically detectable marginal gap formation, or discolorations, are the most relevant outcome parameters, they do not provide information on the integrity of the entire composite-tooth interface and marginal gaps, which are in some instances clinically not detectable, eg, in subgingival areas.

In this respect, additional diagnostic methods might offer information about these parameters. Optical coherence tomography (OCT) is a non-invasive approach to detect insufficiencies even at a subclinical stage and can be applied in clinical studies on adhesives.^16,19,24^ In conventional clinical studies, mainly the restoration margin is assessed. However, it is still unclear to what extent marginal gap formation allows conclusions to be drawn about the integrity of the internal composite-tooth bond.^3^ While both in vitro and in vivo studies using OCT have been conducted on the integrity of the composite-tooth interface and marginal gap formation by application of self-etch and etch-and-rinse adhesives,^3,8,16,19,20,23^ long-term results assessed with OCT are rare.^25^ However, the clinical performance of the two different classes of adhesives, especially over a prolonged period, would be of high practical relevance.

Accordingly, the current study aimed to compare a one-step self-etch and a two-step etch-and-rinse adhesive using OCT to examine the entire internal composite-tooth bond and marginal gap formation, separately for enamel and dentin/cement at 36 to 48 months after restoration placement. These results should be interpreted regarding the clinical outcome data which have already been published.^11^ This previous study found a higher clinical failure rate when using the two-step etch-and-rinse adhesive compared to the one-step self-etch adhesive.^11^ The null hypotheses of the current study are: 1. that the internal composite-enamel bond and the marginal integrity in enamel are superior with the etch-and-rinse adhesives, and 2. the internal composite-dentin interface and marginal integrity in the dentin are superior using the self-etch adhesive vs the etch-and-rinse adhesive. A restorative system based on a proven four-step etch-and-rinse adhesive served as a reference when evaluating both the clinical results in the groups and the bond failure using OCT.

## Materials and Methods

### Study Design

The current investigation was executed as an observational study that examined patients 36 to 48 months after placement of composite restorations with different adhesives. Two adhesives were used with the same composite (comparison of adhesives), and a third adhesive served as a reference in combination with the composite of the product chain (comparison of filling systems). The study was reviewed and approved by the local ethics committee. All patients were informed verbally and in writing about the study and gave their written informed consent.

### Patients

The patients included were participants in a previous randomized clinical trial^11^ and were examined using OCT to evaluate the internal interfacial and marginal gap formation of the placed restorations. Accordingly, the mandatory condition for participation was that patients had received two or three cervical class V composite restorations. The inclusion criteria before restoration placement were positive vitality of the pulp, physiological relationship to natural dentition, as well as a noncarious cervical lesion. Patients with fewer than 20 teeth, heavy bruxism, allergies to tested materials, or examined teeth with contact to removable dentures were excluded.

### Groups and Materials

Three groups were formed for examination (Table 1). As adhesives, the one-step self-etch adhesive Futurabond M (Voco; Cuxhaven, Germany; group SE) and the two-step etch-and-rinse adhesive Solobond M (Voco, group ER) in combination with the nanohybrid composite Amaris (Voco) were used. If patients had a third cervical lesion that needed restorative treatment, a further restoration with the four-step etch-and-rinse adhesive Syntac classic in combination with Tetric EvoCeram (Ivoclar Vivadent; Schaan, Liechtenstein) was placed as a control. In the previous RCT, three experienced operators placed 110 randomly assigned restorations in 40 patients. All operators were dentists in the Department of Cariology, Endodontology and Periodontology, had worked as dentists for at least three years, and had extensive experience in restorative dentistry. A comprehensive description of the randomization and restoration placement procedure is presented in the previous publication.^11^ All teeth were caries-free anterior teeth or premolars, with moderately deep noncarious cervical defects. For cavity preparation, only the upper surface was finished to remove sclerosized dentin, and cavities were cleaned using a fluoride-free polishing paste.

**Table 1:** Groups and materials in self-etch (SE), etch-and-rinse (ER) and control group

Group	Acid	Adhesive/composition (Lot No.)	Composite/composition (Lot No.)
SE	–	Futurabond M/ UDMA, HEMA, BHT, phosphorylated monomer, catalyst, ethanol, water (V35565)	Amaris/ Bis-GMA, UDMA, TEG-DMA, glass ceramic fillers, pre-polymerized ISO fillers, silica nano- particles (760913)
ER	Phosphoric acid 35%, Vococid (0816114)	Solobond M/ Bis-GMA, HEMA, BHT, acidic adhesive monomer, catalyst, acetone (0819366)	
Control	Phosphoric acid 37%, Total Etch (J22302)	Syntac Syntac Primer/ TEG-DMA, PEG-DMA, maleic acid, acetone, water (K49447) Syntac Adhesive/ PEG-DMA, glutaraldehyde, maleic acid, water (L21704) Heliobond/ Bis-GMA, TEG-DMA (J1488)	Tetric EvoCeram Bis-GMA, UDMA, DMDMA, BA-glass, YbF_3_, mixed oxides, pre-polymerized fillers (K43770)

UDMA: diurethane dimethacrylate; HEMA: hydroxyethyl methacrylate; BHT: butylhydroxytoluole; bis-GMA: bisphenol-A glycidylmethacrylate, TEG-DMA: triethylene glycol dimethacrylate; PEG-DMA: polyethyleneglycol dimethacrylate.

Of the patients who were initially treated, 27 (mean age 44.5 ± 16.3 years) with 63 restorations were randomly selected and included in the current observational OCT examination, which was performed independently of the investigations of the previous RCT at a time point between 36 and 48 months after restoration placement (mean 37.9 months). The 63 restorations were distributed as follows: 25 teeth had been restored using Futurabond M/Amaris (SE), 20 with Solobond M/Amaris (ER), and 18 with Syntac classic/Tetric EvoCeram (control).

### Optical Coherence Tomography

A detailed description of OCT, the equipment used, and the standardized recording conditions is given in previous studies by our working group.^19,24^ In this study, the composite restorations were imaged with spectral-domain OCT (SD-OCT, Telesto II SP5, Thorlabs; Dachau, Germany). At the center wavelength 1310 nm, the restored tooth surfaces were scanned point-by-point and line-by-line with the beam of the wide-band light source (spectral bandwidth ± 107 nm) in a maximum field of view of 10 mm x 10 mm x 3.5 mm. Further technical specifications were: axial/lateral resolution < 7.5 (air) µm/15 µm, field of view and depth maximum 10 mm x 10 mm x 3.5 mm, imaging speed 76 kHz, sensitivity ≤ 106 dB and A-scan average of 1.

#### Image acquisition

An experienced, blinded, and calibrated dentist under standardized conditions at the examination appointment performed the OCT recording. The operator-stabilized probe was positioned at a right angle to the surface of the restoration at a distance of 30 to 35 mm, and a 3D OCT image with 150 to 500 cross-sectional images (B-scans) per image stack was generated and exported (ImageJ 1.51 s, National Institute of Health; Bethesda, MD, USA), depending on the mesial-distal extent of the restoration.

#### Gap signal (interfacial adhesive defect)

**Fig 1:**
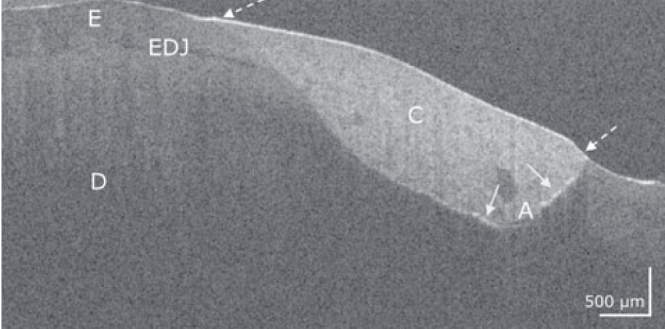
Composite restoration on a premolar with Solobond M/Amaris. In the OCT cross-sectional image, the bond to the composite is intact both at the enamel (E) and dentin (D) margins of the restoration (dotted arrows). Cervical at the dentin-composite (C) interface, two short interfacial adhesive defects appear (bright lines, arrows). An adhesive layer (A) is evident at some points. EDJ: enamel-dentin junction. Scale bars are related to refractive index n = 1.0. While the horizontal scale in the OCT cross-sectional image is independent of the refractive index (n) of the tooth structures, the length of the vertical scale has to be divided by it (mean n for enamel and dentin approximately 1.5).

**Fig 2:**
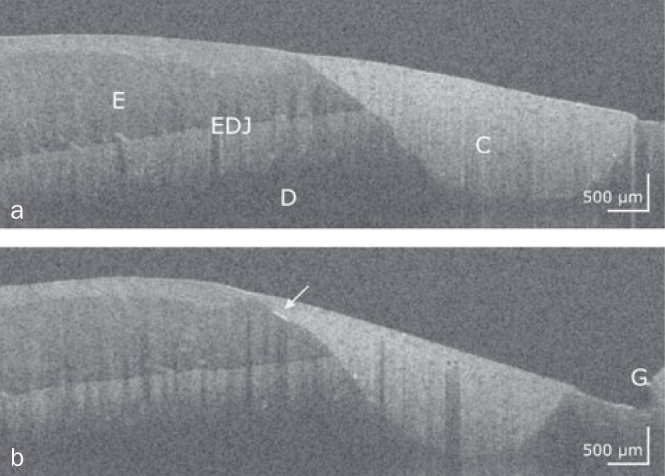
Composite restoration on a front tooth restored with Solobond M/Amaris. Two OCT cross-sectional images of adjacent zones of the composite restoration (C) with intact bond along the entire composite-tooth interface (a) and an internal interfacial gap at the enamel (b, bright line, arrow). E: enamel, D: dentin, G: gingiva, EDJ: enamel-dentin junction. Scale bars are related to refractive index n = 1.0.

**Fig 3:**
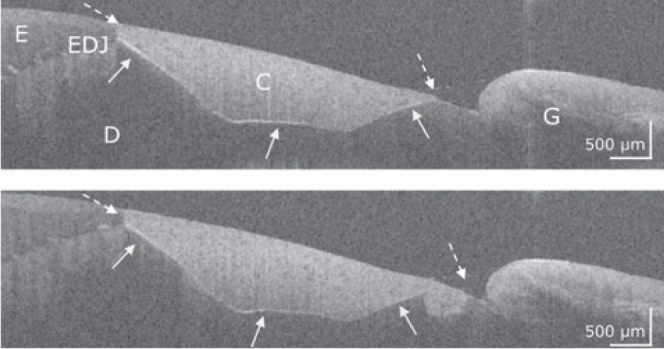
Premolar, restoration system Futurabond M/Amaris. The two OCT cross-sectional images from adjacent zones of the composite restoration (C) show intact restoration margins on enamel (E) and dentin (D) (dashed arrows) but also extended interfacial adhesive defects on dentin (bright lines, white arrows). G: gingiva, EDJ: enamel-dentin junction. The scale bars again refer to the refractive index n = 1.0.

**Fig 4:**
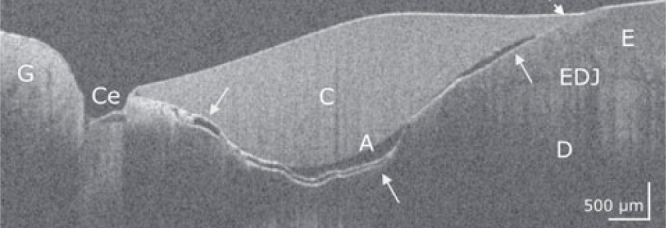
Premolar with restoration system Syntac/Tetric EvoCeram. In the OCT cross-sectional image, the bond to the composite (C) is only intact at the enamel margin of the restoration (dotted arrow). On the cervical margin of the restoration and almost along the entire interface to dentin (D) and enamel (E), a continuous interfacial adhesive defect is evident (bright line, white arrows). A wide gap with embedded material appears at the cervical restoration margin, as does a cohesive defect in the cement (Ce). The adhesive layer (A) is irregularly formed. EDJ: enamel-dentin junction, G: gingiva. Scale bars are related to the refractive index n = 1.0.

All image analytical work was performed on the same monitor under standardized conditions by another trained, blinded, and calibrated examiner instructed in the methodology of image evaluation. A signal for an interfacial adhesive defect (gap) at the restoration margin or at the internal composite-tooth interface is represented by a cluster of pixels with increased brightness compared to the image background (bright line), defined by rising and falling slopes in the A-scans.^19^ Figures 1 to 4 show representative OCT cross-sectional images of the restoration systems with different patterns of interfacial gaps, both at the restoration margin and internally beneath the composite restoration.

#### Analysis of proportion of marginal gap

To determine the proportion of marginal gap per restoration, all B-scans of the restoration were used to capture the entire restoration margin. The number of B-scans was determined for the marginal areas without and with gaps, respectively, and the percentage of images with marginal gaps was determined (ImageJ 1.51).

#### Determination of proportion of internal interfacial gap

Per restoration, the proportion of length for an internal adhesive defect was determined for the individual enamel or dentin/cement-composite interface on 31 equally distributed B-scans of the whole 3D dataset. The percentage for length of adhesive defect on the enamel or dentin per restoration was determined as follows: total length of defect signals ÷ total length specific interface x 100, yielding %.

For analysis, dentin and cementum interfaces were considered together as dentin, since the occurrence of cement, which was clearly detectable in OCT scans, was rare (Fig 4).

### Statistical Analysis

The statistical analysis was performed with SPSS 25.0 for Windows (IBM SPSS Statistics; Armonk, NY, USA). If restorations were lost prematurely, data imputation was performed using the highest rate of gap formation in each group. The mean values (SD) for gap within the groups were calculated. The comparison between groups was performed with Friedman and Wilcoxon tests for dependent variables (values not generally normally distributed, Kolmogorov-Smirnov test). The significance level was set at α = 0.05. In a previous study measuring adhesive defects by OCT, an intrapersonal standard error between 7.0% and 7.2% was determined, depending on the extent of the lesion.^25^

## Results

### Characteristics of Composite-Tooth Bond Failure

Intact and defective restoration margins appeared with very differently developed internal interfacial gaps. It was apparent that marginal gap formation was not necessarily accompanied by extensive internal interfacial gap formation and vice versa. Figures 1 to 4 show typical configurations of this composite-tooth bond failure.

### Quantitative Margin Analysis (Table 2, Fig 5)

**Table 2:** Marginal gap formation at enamel, dentin/cement, and at the entire interface (enamel + dentin/cement, %) for the groups, expressed as means (SD) and medians (Q25/75)

	Enamel			Dentin/cement			Enamel + dentin/cement		
	FBM	Syn	SBM	FBM	Syn	SBM	FBM	Syn	SBM
Mean	35.7	41.5	36.3	50.5	78.0	81.4	42.1	57.0	55.2
SD	23.8	24.0	21.3	24.0	27.4	25.4	19.1	17.2	23.4
Median*	29.9	40.9	33.0	**47.9** ^1,2^	**84.8** ^1^	**92.8** ^2^	**34.3** ^3,4^	**58.7** ^3^	**57.0** ^4^
Q 25%	19.2	22.8	16.5	32.4	64.5	66.2	28.5	45.5	36.2
Q 75%	50.7	62.4	63.6	72.6	100.0	100.0	52.8	65.1	81.6

Significant group differences (p < 0.05) are highlighted in bold. ^1^p = 0.001; ^2^p < 0.001; ^3^p = 0.036; ^4^p = 0.02. *The medians were additionally given since the mean values do not fully describe the distribution due to data imputation (groups SBM, Syn). FBM: Futurabond M; Syn: Syntac; SBM: Solobond M.

**Fig 5:**
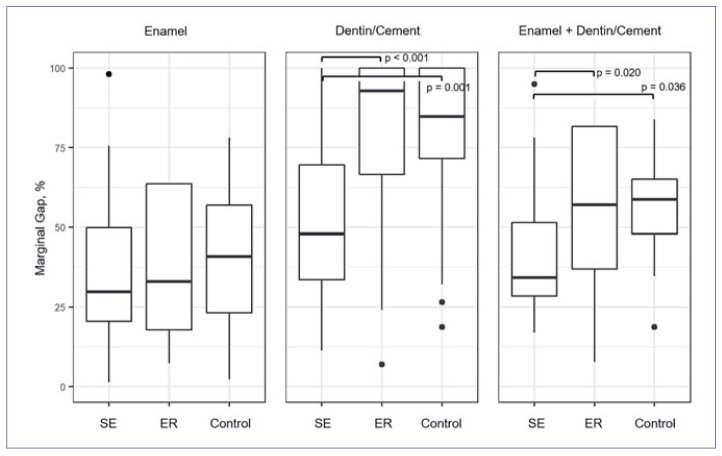
Boxplot (median, quartiles 25%/75%) of marginal gap formation at enamel, dentin/cement and at the entire interface (enamel + dentin/cement, %) for the groups. Significant group differences are marked (p < 0.05).

In enamel, the percentages of gap formation between the groups were not significantly different (index p-value: p_i_ ≥ 0.258). In dentin/cement, SE showed significantly less gap formation compared to ER (p < 0.001) and control (p = 0.001). In group SE, when merging enamel and dentin/cement-supported areas, significantly lower percentages of gap formation than in groups ER (p = 0.020) and control (p = 0.036) were observed.

### Internal Composite-Tooth Bond Failure (Table 3, Fig 6)

**Table 3:** Internal interfacial gap formation at enamel, dentin/cement and at the entire interface (enamel + dentin/cement, %) for the groups, expressed as means (SD) and medians (Q25/75)

	Enamel			Dentin/cement			Enamel + dentin/cement		
	FBM	Syn	SBM	FBM	Syn	SBM	FBM	SYN	SBM
Mean	25.1	23.9	29.1	20.1	47.6	55.6	21.4	39.5	49.3
SD	16.7	26.2	26.8	16.0	32.7	35.3	15.1	26.6	42.4
Median*	21.4	14.6	20.0	**16.9** ^1,2^	**45.9** ^1^	**61.6** ^2^	**18.3** ^3,4^	**41.1** ^3^	**50.7** ^4^
Q 25%	11.5	6.1	5.3	7.6	18.2	12.3	9.6	17.1	14.8
Q 75%	33.4	31.5	67.6	24.5	77.1	94.3	26.1	68.5	87.6

Significant group differences (p < 0.05) are highlighted in bold. ^1^p = 0.003; ^2^p < 0.001; ^3^p = 0.008; ^4^p = 0.001. *The medians were additionally given since the mean values do not fully describe the distribution due to data imputation (groups SBM, Syn). FBM: Futurabond M, Syn: Syntac, SBM: Solobond M.

**Fig 6:**
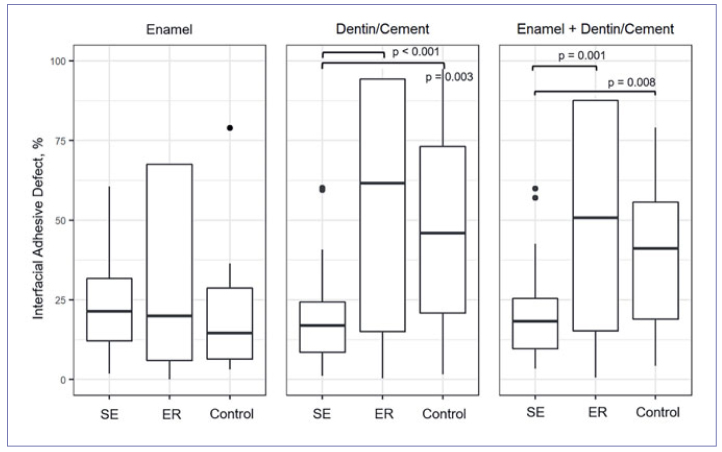
Boxplot (median, quartiles 25%/75%) of internal interfacial gap formation at enamel, dentin/cement and at the entire interface (enamel + dentin/cement, %) for the groups. Significant group differences are marked (p < 0.05).

The percentages of the lengths for enamel and dentin/cement at the composite-tooth interface in the groups were 14% and 86% for SE, 24% and 76% for ER, and 23% and 77% for control, respectively.

At the composite-enamel interface, the percentages of adhesive defects between the groups were not significantly different (p_i_ ≥ 0.747). At the cement-composite/dentin interface, the SE group showed significantly fewer adhesive defects compared to ER (p < 0.001) and control (p = 0.003). Values for ER and control were not significantly different (p = 0.898). For the entire composite-tooth interface, the SE group showed significantly fewer adhesive defects than did the ER (p = 0.001) and control groups (p = 0.008); again, the values for ER and control were not significantly different (p = 0.648).

## Discussion

In the current study, a comparison was made between the adhesives Futurabond M (SE) and Solobond M (ER) and, when considering the control group, a comparison of the restoration systems Futurabond M/Amaris, Solobond M/Amaris, and Syntac/Tetric EvoCeram was made.

While the first hypothesis (superiority of ER in enamel) could not be verified, the second hypothesis (superiority of SE in dentin) was confirmed. At the predominant composite-dentin interface, the self-etch adhesive Futurabond M was significantly more effective than the etch-and-rinse adhesive Solobond M, both at the restoration margin and the internal composite-tooth bond. This also applies when the enamel, dentin, and cement were considered together. Thus, the question posed, whether a statement about the entire composite-tooth interface is possible based on the restoration margin, can be answered affirmatively for this study. The OCT-based results are in line with those of the clinical comparison of the two adhesives by this working group, as after three years of clinical service, the etch-and-rinse adhesive resulted in a significantly higher cumulative failure rate (33.3%) than did the self-etch adhesive (9.1%).^11^ The clinically detectable higher rates of small marginal defects and chip fractures on dentin compared to enamel in the ER group^11^ are also consistent with OCT in terms of absolute values for marginal and internal gap formation (Tables 2 and 3, Figs 5 and 6).

The non-destructive OCT method provides insight into both the marginal and the internal composite-tooth adaptation.^24^ Marginal defects in OCT are in line with those seen on replicas of restorations using scanning electron microscopy (SEM) and can be related to restoration loss.^3,25^ It was demonstrated that the technique can reveal subtle interfacial adhesive defects on the enamel and dentin that may interfere with clinical success.^8,9^ Accordingly, OCT has recently been applied in different in vitro and clinical studies with a focus on the performance of adhesive strategies regarding the marginal and internal adaption of composite and can be seen as a valid method.^2,3,8,9,19,20^

The comparison of adhesive strategies is still discussed controversially, with recent meta-analyses on this topic.^15,26,29^ In general, these analyses confirm clinically satisfactory performance of SE adhesives, while merely clinical outcome parameters were considered. Schroeder et al^26^ summarized that the retention rates would be comparable between different adhesive strategies, but ER procedures would result in less discoloration at enamel margins.^26^ This is also supported by recent results on the clinical performance of a universal adhesive in self-etch mode.^22^ With OCT, marginal discolorations are not verifiable. However, it is possible to assess only marginal gap formation or structures such as excess material at the restoration margin, marginal fractures, or cracks in the restorative material, which may be combined with non-removable, unacceptable marginal discolorations. In the present study, the marginal gap was evaluated in this respect. The fact that there were no significant differences between the two adhesive strategies for the enamel margin does not contradict the 3-year clinical results of the study. Indeed, with regard to the esthetic criteria, all restorations in both groups were clinically acceptable after three years.^11^ In other words, the significant differences in the marginal gap on dentin were not clinically reflected in the criterion marginal discoloration.

In the current study, the superior results of SE adhesive for marginal and internal adaption in dentin are conspicuous. In principle, the improved ability of SE to maintain dentin hydroxyapatite while avoiding collagen collapse in dentin tubules might be beneficial for the formation of a sufficient bond to composite.^7^ Ozer et al^17^ concluded that SE adhesives would provide superior and more predictable bond strength to dentin and thus derived the recommendation to use SE adhesives in cavities predominantly located in dentin. Accordingly, the results of this study support the beneficial properties of the SE adhesive for cervical dentin-supported restorations. However, a recent randomized trial demonstrated increased dentin bond strength if SE or universal adhesives were combined with phosphoric acid etching in etch-and-rinse or selective enamel-etching mode.^18^ Specifically, in vivo studies using OCT provided information about the increased integrity of the composite-dentin interface (sclerotic dentin) by using a universal adhesive.^8,9^

Another aspect was comparing marginal and internal interfacial adaptation of the composite to enamel using the adhesives ER and SE. In this study, no significant differences were found for the SE and ER adhesives. In contrast, a recent study using SEM demonstrated the superiority of the ER procedure in enamel.^4^ Also, Frankenberger and Tay^6^ reported more effective enamel bonding of an ER adhesive, and another 36-month clinical study reported more clinical enamel margin degradation for SE adhesive.^13^ Likewise, previous studies by this working group found more adhesive defects at the enamel-composite interface for SE mode at 6 or 12 months after placement.^8,9^ Accordingly, ER systems are regularly preferred when cavity margins show a large amount of enamel.^17^ Nevertheless, a recent study which examined the application of universal adhesives in SE mode showed satisfactory performance in an in vitro setting.^28^ This was also the case in the present study, and some aspects can be discussed to explain these findings. First, every adhesive material has a distinct behavior and different ability to bond to enamel and dentin.^2^ In long-term clinical performance, wide variations exist, independent of the adhesive strategy.^30^ For example, the adhesive Futurabond M contains a phosphorylated methacrylate, whose chemical activity appears comparable to that of 10-MDP, and thus an effective chemical bond to the enamel and hypermineralized dentin can be assumed.^11^ Another factor is the proportion of enamel and dentin at the composite-tooth interface. It is known that ER systems are superior when large enamel-supported areas exist within the cavity.^17^ In the current study, the enamel:dentin ratio was 1:6 in the SE group. Accordingly, when using the SE adhesive, the significantly more extensive composite-dentin interface could compensate for the possibly less strong bond at the enamel in this group. This is supported by the fact that the differences between SE and ER groups did not change, and the superiority of the self-etching adhesive was maintained, when enamel and dentin were combined.

When comparing the three restoration systems on dentin by OCT, the Futurabond M/Amaris system was superior to both Solobond M/Amaris and Syntac/Tetric EvoCeram. This differs from the clinical results, in which retention rates after three years were significantly higher in both the SE and control groups than in the ER group. Nevertheless, in agreement with the OCT, the restorations in the ER and control groups clinically showed significantly more small defects in dentin than at the enamel margins. Therefore, the significantly increased bond strength at the composite-tooth interface in the SE group compared to the control group (OCT) was not clinically evident after three years. This may indicate higher sensitivity of OCT compared to clinical assessment; it is consistent with the results of previous clinical trials, in which OCT was performed in parallel and indicated clinical failures in advance.^8,9^ This suggests that observation periods of three years are too short to evaluate the long-term performance of composite restorations. At least five years seem to be needed as a clinical observation period to detect carious lesions at restoration margins, which would then require restoration replacement.^3^ In addition, a direct comparison of the two groups, SE and control, is complicated because the comparison refers to restoration systems in which both adhesives and the restoration materials are different.

The special feature of the current study is the use of OCT for non-invasive assessment of the composite-tooth bond of Class V restorations in situ compared to their clinical evaluation. The period of 3 to 4 years since restoration placement allows conclusions to be drawn about the clinical performance of the adhesives or restoration systems. It complements the data in the literature, where primarily in vitro data and few results obtained with OCT in vivo are available.

Although OCT imaging of the restorations and image analysis were performed in a blinded and standardized setting, limitations in the clinical approach must be mentioned. These include the placement of the restorations by three practitioners and the heterogeneity of the defects, as well as the lack of OCT data immediately after placement of the restorations. The initial data could serve as a reference, especially since data imputation was performed in case restorations were lost. Therefore, the groups’ mean values for marginal gap and internal interfacial gap are presented in a modified form. Differences in cavity size, depth, and enamel:dentin ratio, which may also influence the results, should have been minimized by randomizing the cavities. Despite these limitations and the fact that only 27 patients and 63 restorations were included in the study, differences between adhesives under 3-year clinical assessment were presented. The discrepancy with the clinical assessment when comparing the three restoration systems could result from the predictive nature of the OCT data, which could become relevant as the lifetime of the restoration increases.^8,9^ In contrast to the assessment with OCT, the clinical examination was performed with 40 patients on a total of 110 restorations. This suggests that the metric parameter “interfacial adhesive defect” provides greater statistical power than the clinical criteria for evaluating adhesives or restoration systems. By analogy, this has already been noted for quantitative marginal analysis based on analysis of replicas with scanning electron microscopy.^10^ Regardless, the reduced sample of 27 out of originally included 40 patients must be considered. There were different reasons for this reduction in the current study: 1. The OCT was an additional examination method, for which patients needed a separate appointment. Therefore, patients needed to agree, give a new consent to participate, and come to the clinic independently of the previous study-related appointments; 2. Only restorations which were assessable in OCT (accessable for the OCT scan, ie, anterior teeth and premolars, if mouth opening was adequate) were included; and 3. only restorations which were still in situ could be assessed. Altogether, this must be seen as a bias in the study sample, limiting the conclusions drawn from the data. Moreover, this study evaluated only one self-etching and one two-step adhesive and, if applicable, a four-step etch-and-rinse adhesive. This study did not consider two-step self-etch adhesives or three-step etch-and-rinse adhesives.

## Conclusions

The self-etching adhesive used in the current investigation showed increased integrity of the margin and internal interfaces in dentin compared to the etch-and-rinse adhesives. Thus, it appears to be recommendable for restorations of noncarious cervical lesions, especially when the cavity surface mainly consists of dentin. The assessment of the bond at the restoration margin and internal adaptation was equivalent.

## Clinical relevance:

The self-etch adhesive FuturaBond M appears recommendable for restoration of cervical class V lesions. OCT can be used for imaging the restorations and evaluating the restoration systems.
